# Optimized Production of Xylanase by *Penicillium purpurogenum* and Ultrasound Impact on Enzyme Kinetics for the Production of Monomeric Sugars From Pretreated Corn Cobs

**DOI:** 10.3389/fmicb.2020.00772

**Published:** 2020-04-24

**Authors:** Bindu Sunkar, Balakrishna Kannoju, Bhima Bhukya

**Affiliations:** Centre for Microbial and Fermentation Technology, Department of Microbiology, University College of Science, Osmania University, Hyderabad, India

**Keywords:** *Penicillium purpurogenum*, corn cobs, solid state fermentation, *Taguchi* design, optimization, xylanase, ultrasound

## Abstract

Corn cob is an abundant organic source with significant potential in sustainable energy development. For the effective conversion of the feedstocks to valued commodities, effective biocatalysts are highly desired. The present study aims at optimizing the critical parameters required for xylanase production by *Penicillium purpurogenum* isolated from rotten wood sample using the *Taguchi* orthogonal array layout of L25 (5^∧^6). The optimized conditions like temperature 40°C, pH 3, size of inoculum 1.2 × 10^8^ spores/ml, moisture 70%, peptone 0.8%, and 5 days of incubation resulted in 1,097 ± 6.76 U/gram dry substrate (gds) xylanase which was 65.72% more when compared to un-optimized production of xylanase. The xylanase thus produced, effectively carried out pretreated corn cob saccharification and the reaction was further improved with ultrasound assistance which has increased the saccharification yield to 12.02% along with significant reduction in reaction time. The saccharification efficiency of pretreated corn cob was found to be 80.29% more compared to the raw corn cob, reflecting its recalcitrance to digestion. Indeed, xylan being the second most abundant polymer in lignocellulosic biomass, considerable attention is being paid for its effective conversion to valued products.

## Graphical Abstract

An overview of xylanase production by *P. purpurogenum* and its application in corn cob saccharification.

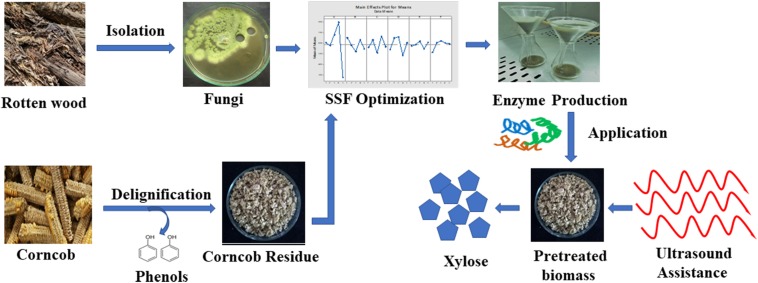

## Introduction

Plant based biomass is the most abundant organic source on the earth and it can be utilized for wide industrial application in the production of varied products. In view of the concern about the cost and yield in the biorefinery industries, an emphasis is laid on the production of cellulases and xylanases. Therefore, production of higher enzyme titers using renewable resources will bring out a major change in the field of biofuels. Agriculture byproducts like corn cobs is one of the most renewable lignocellulosic feedstocks produced from corn processing industry (Kumar et al., 2008) with significant level of hemicellulose content. According to Knob et al. (2014), for 100 kg of corn grains, 18 kg of cobs are produced which is either discarded or sold at low price for animal supplementary feed (Ashour et al., 2013) which is notwithstanding its potential. The utilization of such abundant agriculture biomass may contribute to increase its value and not worsen environmental pollution (Kumar et al., 2017; Pandiyan et al., 2019).

Xylanases are a set of enzymes that act on the hemicellulolytic part of lignocellulose and ultimately result in xylose, the second most abundant carbohydrate after glucose. Xylan is a polymer with D-xylopyranosyl residues linked with β-1, 4 linkage (Khusro et al., 2016) which is thermo and acid labile. Therefore application of severe conditions for the hydrolysis of hemicellulose, would result in the formation of undesirable inhibitors. Hence, enzyme saccharification would be a great choice of hydrolysis for hemicellulose to avoid toxic inhibitors. Apart from biorefinery, xylanases have a potential industrial application in various fields like wine making, textile, paper and pulp industries, animal husbandry, bread making, fruit juice extraction (Chadha et al., 2019) etc. Production of xylanases in large scale is gaining much importance in getting the scope to cut down the enzyme cost with the use of high yielding microbes (Ramanjaneyulu and Rajasekhar Reddy, 2016). Xylanase production by various organisms have been studied like fungi (Chadha et al., 2019), bacteria (Kumar et al., 2018), yeast, (Ergun and Çalık, 2016), and even with co-culture of more than one organism (Yardimci and Cekmecelioglu, 2018). Among them, fungi have been found to be potent xylanase producers and therefore many filamentous fungi have been reported for the production of xylanase. Various species of *Trichoderma* and *Aspergillus* (Pal and Khanum, 2010) have been extensively studied and reported to produce functionally diverse xylanases. *Penicillium purpurogenum* was also found to produce xylanases of different isoforms (Belancic et al., 1995) and their patterns were identified by Gonzalez-Vogel et al. (2011) who found different xylanases in the culture medium based on the carbon source used. Some of the proteins like endoxylanases (Chávez et al., 2002), β-glucosidase (Hidalgo et al., 1992), acetyl xylan esterases (Egana et al., 1996; Gordillo et al., 2006), and arabinofuranosidases (De Ioannes et al., 2000; Fritz et al., 2008; Ravanal et al., 2010) required for the lignocellulose digestion have been purified and characterized for their potential application in industries. Many investigators have reported on xylanases produced from *Penicillium* species (Table 1).

**TABLE 1:** Xylanase production by different *Penicillium* species from previous study.

Organism	Substrate used	Methodology	References
*Penicillium funiculosum*	Cellulose and wheat bran	SMF	Mishra et al., 1985
*Penicillium canescens* 10*-*10c	Wheat straw with xylan	SSF	Bakri et al., 2003
*Penicillium brasilianum* IBT 20888	Mixture of cellulose and xylan	SMF	Jorgensen et al., 2003
*Penicillium janthinellum*	Corncob, corn husk, oat husk, and bagasse	SMF	Oliveira et al., 2006
*Penicillium schelotiorum*	Oat spelt xylan and wheat bran	SSF	Knob and Carmona, 2008
*Penicillium janczewskii*	Brewer’s spent grain	SMF	Terrasan et al., 2010
*Penicillium oxalicum* GZ-2	Wheat straw	SMF	Liao et al., 2012
*Penicillium echinulatum*	Sorbitol and cellulose	SMF	Todero et al., 2013
*Penicillium chrysogenum*	Sugar cane bagasse	SSF	Terrone et al., 2018

Industrially, purified xylan is used as substrate which is found to induce the production of xylanase is uneconomic, and therefore using the substrates rich in xylan are more suitable for the production of xylanase (Bhardwaj et al., 2019). It is also reported that acetylated xylan acts as a good inducer in expressing different patterns of xylanase by *P. purpurogenum* (Navarrete et al. (2012). Corn cobs contain ∼40% xylan which is highest amount among the other lignocellulosic feedstocks, thereby making it a potential substrate for xylanase production (Knob et al., 2014). Application of enzymes in various industrial fields and their importance in research has already aroused. The main drawback in biocatalytic approach is the hydrolysis time which is too high and therefore requires high loads of enzymes especially in its crude form. Therefore, it is essential to enhance the enzyme activity where ultrasound plays a potential role by reducing the reaction time (Sun et al., 2019).

Solid state fermentation (SSF) is a technique where a solid matrix is used as both physical support as well as nutrient source for the growth of organism which require little or no free moisture (Pandey et al., 2000). Under the SSF, the microorganisms grow in conditions close to natural habitat, therefore more capable of producing enzymes compared to submerged fermentation (SMF) (Jecu, 2000). SSF is advantageous over submerged for low capital cost, higher productivity, higher end product stability and easy downstream processing (Singhania et al., 2008). However, both SSF and SMF have their own pros and cons because of difference in metabolism of the organism that require specific parameters which otherwise may lead to an undesirable product (Subramaniyam and Vimala, 2012).

Either in case of SSF or SMF, several factors have their significant role and impact on final yield and productivity. The critical factors that have shown to possess greater impact on overall process and yield are substrate to moisture ratio, pH, temperature, inoculum size and the nutrient source which need to be optimized for higher productivity (Bhardwaj et al., 2019). The conventional optimization method involves changing of each variable at a time, keeping the others constant, that does not consider the interaction effects between the variables (Kannoju et al., 2017). However, all the parameters are interlinked and may result differently when each factor was studied independently or in combination with other parameters. Therefore conventional approach with optimization of one factor at a time (OFAT), may not be reliable (Adewale et al., 2017). *Taguchi* methodology is one of the ideal optimization tools for designing factorial experiments that provides a systemic plan for the experiments to be performed and also provides the correlation between the parameters when applied with different combinations. *Taguch*i method of orthogonal array gives an extensive data with minimum experimental runs. These statistical tools facilitate to identify the impact of individual factor, factors in combination and finally establish the overall performance at the optimum conditions with the minimum experimental setups.

Microflora of different natural niches play an important role in maintaining the biogeochemical cycles and therefore needs to be explored for the identification of potential microbes of commercial importance. In nature, wood degrading basidiomycetes play a critical role in degrading the cellulose and hemicellulose with the ability to produce a wide range of digestive enzymes (Couturier et al., 2018). In this context, the present study aims at isolating the potential fungal strain from the diverse rotten wood samples and screening for the production of extracellular xylanase by submerged fermentation. The obtained isolate was subsequently used for the production of xylanase by evaluating the optimum conditions for solid state fermentation. The enzyme extract was evaluated for the saccharification potential using pretreated corn cob as substrate. The hydrolysis process was improved with ultrasound and its significance in enzyme activation has been evaluated.

## Materials and Methods

### Substrate for Xylanase Production

De-seeded corncobs were collected from various localities of Hyderabad, Telangana State, India. The substrate was initially sun dried and were subjected to oven drying to make them moisture free. The substrate was then milled and sieved to get a uniform particle size of 2–3 mm and ground substrate was used for the study. The chemical composition of the substrate was determined by standard TAPPI (Technical Association of Pulp and Paper Institute [TAPPI], 1992) methodology.

All the chemicals used in the experiment were of analytical grade (Merck & Co., Inc., Germany). The standard, oat spelt xylan used as the substrate for the enzyme assay was purchased from Sigma-Aldrich Pvt. Ltd., United States.

### Delignification

Pretreatment of biomass was carried out by taking 20 g of substrate in a 500 ml Erlenmeyer flask added with 2% (w/v) NaOH at 1:10 ratio and allowed to react for 4 h at 50°C. Contents were filtered through muslin cloth and the biomass was washed under running tap water till neutral pH. The filtrate was analyzed for phenolic compounds and the leftover residue was oven dried for 12 h at 60°C and again, the composition analysis was done to know the impact of delignification. Further, the delignified residue was used as the sole carbon source for the subsequent fermentation and saccharification studies.

### Isolation of Fungi and Method of Cultivation

A wide variety of rotten wood samples were collected from different places of Telangana State, India for the isolation of extracellular xylanase producing fungi. In a 100 ml Erlenmeyer flask, 1 g rotten wood sample was suspended in 25 ml sterile distilled water and kept in a rotary shaker at 30°C for 2 h. After incubation, the samples were serially diluted and 50 μl of sample was inoculated on to the potato dextrose agar (PDA) medium plates containing kanamycin (50 μg/ml) and incubated at 30°C for 5 days. After incubation, the fungal colonies were sub cultured on PDA slants and preserved for further studies.

### Screening of Xylanase Producing Fungi

#### Primary Screening

All the fungal isolates were primarily screened for the xylanase producing ability by Congo red assay as described by Teather and Wood (1982). Fungal plugs (5 days old) were inoculated on PDA agar plates supplemented with 1% (mass basis) oat spelt xylan and incubated at 30°C for 3 days. Then plates were flooded and incubated with 1.0% Congo red for 15 min. Subsequently, plates were destained using 1 M NaCl for 30 min. Positive strains were identified by a clear zone of hydrolysis around the xylanase producing strains. Based on above assay, strains were selected and used for secondary screening.

#### Secondary Screening

The selected fungal isolates were further screened by submerged fermentation using delignified corncob residue as the sole carbon source. Submerged fermentation was carried out in 100 ml mineral salt medium containing (g/L) peptone 5.0, KH_2_PO_4_ 0.3, K_2_HPO_4_ 0.3, NH_4_SO_2_ 0.5, MgSO_4_.7H_2_O 0.3, and CaCl_2_ 0.3 along with 2% delignified corn cob substrate in a 250 ml Erlenmeyer flask and sterilized at 121°C for 15 min. The flasks were then cooled and inoculated with 3 fungal plugs of 5 days old culture and incubated for 7 days at 30°C and 120 rpm. Samples were collected at every 24 h interval from each flask in a 2 ml Eppendorf tube and centrifuged at 10,000 rpm for 10 min. The supernatant thus obtained was used as crude enzyme and the xylanase activity was expressed as U/ml. Based on the activity values obtained from the enzyme assay, the potent strain which produces relatively higher xylanase was selected and preserved for further studies.

#### Molecular Characterization

The fungal isolate PF7 was selected as best xylanase producer and characterized by sequencing its 5.8S rRNA using the 18S rRNA sequencing platform of Macrogen Inc., South Korea. Complete sequence of 5.8S rRNA was BLAST searched in NCBI database^1^ to find the sequence similarity for the organism. Based on sequence similarity, the organism was characterized and the sequence was submitted to NCBI Genbank. Further, phylogenetic dendrogram was constructed based on evolutionary history using Neighbor-joining method of MEGA X software. Percentage of replicate trees associated with taxa clustered together with 1000 bootstraps was performed. The evolutionary distances were computed using Jukes–Cantor method (Huelsenbeck, 1995).

### Parameters Optimization by *Taguchi* Methodology

#### Experimental Design

*Taguchi* experimental design was set up to optimize the critical factors that influence the production of extracellular xylanase using solid state fermentation by *P. purpurogenum.* According to the design, a set of arrays with 25 experimental runs were carried out using standard orthogonal array L25 (5^6^). A total of 25 experiments were performed with different combinations to evaluate the effect of each parameter on the production of xylanase by the strain PF7. The critical factors selected for optimization were temperature, pH, moisture level, inoculum size, peptone concentration, and incubation time. Each factor of six, was assigned with five levels as shown in the Table 2. For all the 25 experimental trials with 6 factors and five levels, the results were expressed in enzyme units per gram dry substrate (Table 3). The influence of each factor on the xylanase production was determined using *Taguchi* approach by Windostat version 9.1. Analysis of variance (ANOVA) was performed to know the impact of each factor at specific level on the final yield of the enzyme.

**TABLE 2:** Factors with assigned levels of experimental design for xylanase production.

Factors	Description	Units	Level 1	Level 2	Level 3	Level 4	Level 5
A	Temperature	°C	25	30	35	40	45
B	pH	pH	3	4	5	6	7
C	Inoculum size	Spore count	0.6 × 10^8^	0.8 × 10^8^	1.0 × 10^8^	1.2 × 10^8^	1.8 × 10^8^
D	Moisture	%	50	60	70	80	90
E	Incubation time	Days	3	4	5	6	7
F	Nitrogen source (peptone)	% (w/v)	0.4	0.6	0.8	1.0	1.2

**TABLE 3:** *Taguchi* orthogonal array of L25 (5^6^) experimental design for xylanase production by *Penicillium purpurogenum*.

Experimental	Levels of parameters used in						Xylanase activity
run no.	the experimental design						(Ugds^–1^) ± SD
	*A*	*B*	*C*	*D*	*E*	*F*	
1	1	1	1	1	1	1	481.30 ± 26.06
2	1	2	2	2	2	2	698.60 ± 20.40
3	1	3	3	3	3	3	604.73 ± 26.91
4	1	4	4	4	4	4	622.63 ± 30.87
5	1	5	5	5	5	5	594.10 ± 23.73
6	2	1	2	3	4	5	705.43 ± 19.82
7	2	2	3	4	5	1	365.00 ± 29.36
8	2	3	4	5	1	2	611.83 ± 22.45
9	2	4	5	1	2	3	589.37 ± 40.31
10	2	5	1	2	3	4	632.93 ± 24.10
11	3	1	3	5	2	4	702.20 ± 24.19
12	3	2	4	1	3	5	746.87 ± 27.44
13	3	3	5	2	4	1	548.00 ± 27.04
14	3	4	1	3	5	2	810.90 ± 23.74
15	3	5	2	4	1	3	605.03 ± 26.72
16	4	1	4	2	5	3	1060.10 ± 30.00
17	4	2	5	3	1	4	848.90 ± 27.61
18	4	3	1	4	2	5	615.63 ± 29.78
19	4	4	2	5	3	1	863.13 ± 32.77
20	4	5	3	1	4	2	615.43 ± 29.04
21	5	1	5	4	3	2	278.93 ± 12.71
22	5	2	1	5	4	3	254.90 ± 34.22
23	5	3	2	1	5	4	247.23 ± 30.45
24	5	4	3	2	1	5	306.33 ± 38.02
25	5	5	4	3	2	1	314.23 ± 34.11

#### Inoculum Preparation

The isolated and screened fungal strain was sub cultured on PDA slants and incubated for 7 days for spore formation. Once the spores were produced, slants were washed with 5 ml solution (0.8% NaCl) and 10 μl of tween 80 was added to decrease the surface tension and to get uniform spore suspension. Spore count was done using Neubauer counting chamber and based on the experimental design, the assigned levels of inoculum size were prepared and used for the optimization experiment.

#### Solid State Fermentation

Experiment was carried out with 5 g of pretreated corn cob residue in 250 ml Erlenmeyer flask and the factors, pH, moisture, peptone concentration and inoculum size were set according to the 5 levels of the experimental design. All the flasks were sterilized at 121°C for 15 min. After sterilization, flasks were brought to room temperature and required amount of spore suspension with five different levels were inoculated according to the experimental design and incubated at different set of temperatures for different incubation periods till 7 days. After incubation, the flasks were harvested for the extraction and evaluation of enzyme production.

### Enzyme Extraction and Assay

To all the incubated flasks, 50 ml of 50 mM sodium phosphate buffer (pH 5.0) was added for the enzyme extraction. Flasks were kept for 60 min at room temperature under gentle shaking. The contents in the flasks were extracted by squeezing through muslin cloth and the obtained filtrate was then filtered through Whatman no. 1 filter paper, and centrifuged at 10,000 rpm at 4°C for 10 min. The supernatant thus obtained was used as the crude enzyme and assay was carried out according to Bailey et al. (1992). Enzyme reaction was carried out using 0.9 ml of 1% oat spelt xylan as the substrate prepared in 50 mM sodium phosphate buffer (pH 5.0) added with 0.1 ml of crude enzyme dilution and incubated at 55°C for 10 min, and the reaction was terminated by adding 2 ml of 3, 5-dinitrosalicylic acid (DNS) and the absorbance was read at optical density (O.D) 540nm (Miller, 1959). The absorbance of the sample was deduced by plotting a graph against the blank. The enzyme activity was defined as one International Unit (IU) of xylanase is the quantity of enzyme that releases 1 μmole of xylose per min under the standard assay conditions. The enzyme activity was expressed in units per gram dry substrate. The xylanase activity thus obtained was the average value for the three independent determinations.

### Analysis of Biomass Consumption

Once the parameters were optimized, experiment was set up with the optimized conditions to investigate biomass consumption during the growth during SSF by *P. purpurogenum*. For the biomass consumption analysis, 7 flasks were maintained for 7 days of incubation and each flask was harvested every day for 7 days. The flasks were harvested by saline (0.8% NaCl) wash and followed by drying at 60°C for 24 h to remove the leftover moisture and weights were noted. Difference in biomass weight gives the actual utilization of biomass by the organism for enzyme production during fermentation.

### Evaluation of Xylanase Efficacy on Corn Cob Saccharification

For the evaluation of enzyme saccharification, both raw and alkali pretreated corn cob substrates were used. The culture filtrate from solid state fermentation was used as the crude enzyme source for biomass saccharification analysis. Saccharification analysis was done in two sets, one with each raw and pretreated substrate, respectively. Enzyme reaction was carried out with 10 g of substrate in a 250 ml Erlenmeyer flask containing 100 ml of 50 mM citrate buffer (pH 4.8) along with crude enzyme solution. Each flask was added with different enzyme concentrations (1,000, 1,500, 2,000, 2,500, and 3,000 U/g substrate), and added with 0.1% (w/v) sodium azide to prevent microbial contamination. A negative control was maintained with heat inactivated enzyme. The reaction was carried out at 50°C in a water bath for 72 h. At every 24 h interval, 2 ml of sample was collected in sterilized Eppendorf tube and centrifuged at 10,000 rpm for 5 min and the obtained supernatant was analyzed for sugars produced using High Performance Liquid Chromatography (HPLC).

### Improvement of Enzyme Saccharification by Ultrasound

To evaluate the impact of ultrasound on enzyme hydrolysis, a separate hydrolysis reaction was carried out with same reaction mixtures as above using pretreated corn cob residue as the substrate for hydrolysis in an ultrasound bath (Soltec Soluzioni Tecnologiche, Luglio, Italy). The sonicator was set to 40 kHZ, power output of 120 W with the reaction temperature 50°C and 60% duty cycles with a reaction time of 8 h. At every 2 h interval, 2 ml of reaction sample was collected, centrifuged at 10,000 rpm for 10 min and the supernatant was evaluated for released fermentable sugars. The results from both the enzyme hydrolysis with and without ultrasound were compared to evaluate the efficiency of ultrasound in enzyme activation and the saccharification efficiency was calculated using the following formula

$$\text{Saccharification (\%)}=\frac{\mathrm{Sugars}\,\mathrm{released}}{\mathrm{Total}\,\mathrm{hemicellulose}\,\mathrm{in}\,\mathrm{biomass}\,\left(\mathrm{pretreated}\,\mathrm{or}\,\mathrm{raw}\right)}\times 100$$

### Analysis of Monomeric Sugars by HPLC

The released sugars during the enzyme saccharification of corn cob (both raw and pretreated) were analyzed by HPLC method as described by Gupta et al. (2012). Briefly, HPLC (Shimadzu, Kyoto, Japan) system equipped with a monosaccharide-zodiac Carb 87C column (300 mm × 7.8 mm), RI detector, LC 20AD PUMP-B, LC-20AD PUMP-A, DGU-20A3 degasser and LC solutions software was used for this study. The column temperature was maintained at 30°C and the flow rate was set to 0.6 ml/min. HPLC grade water was used as solvent after filtering and degassing by inline degasser. HPLC run for each sample was carried out for 15 min followed by the column equilibration with HPLC grade water and the peaks were detected at 210 nm. The results were quantified and expressed based on the xylose and glucose standards (Sigma-Aldrich) with a concentration of 1 mg/ml.

## Results

### Substrate, and Its Delignification

Corn cobs contain 34.7 ± 0.48, 33.8 ± 0.64, and 18.4 ± 0.32% of cellulose, hemicelluloses and lignin, respectively. Due to its high lignin content, the biomass was pretreated prior to solid state fermentation with 2% NaOH which resulted in 84.7% delignification. The composition of delignified corn cob residue was found to be 33.8 ± 0.76, 31.08 ± 0.92, and 2.72 ± 0.76% of cellulose, hemicellulose and lignin, respectively. The removal of lignin leads to break down of hetero polymer complex and thereby increases the surface area of the biomass for the enzymes to act on.

### Isolation of Fungi

A total of seventy-nine fungal stains were isolated from various rotten wood samples aiming to get a potent strain capable of producing extracellular xylanase in particular.

### Screening for Xylanase Producing Fungi

**FIGURE 1:**
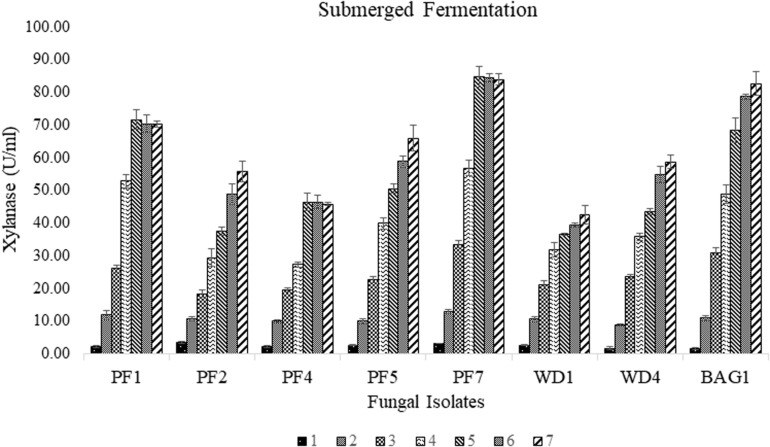
Secondary screening of fungal isolates by submerged fermentation using corncob as sole carbon source for xylanase production (U/ml) with respect to incubation time (days).

Among the seventy-nine strains, eight strains (PF1, PF2, PF4, PF5, PF7, WD1, WD4, and BAG1) were found to be xylanase positive for the Congo red plate assay using xylan as substrate. Large transparent hydrolyzed zones were produced around the fungal colonies with xylanase production. The selected strains were further screened for the efficiency of xylanase production under submerged fermentation using delignified corncob as the sole carbon source. Among all the selected strains, PF7 was found to be the most promising strain with significantly (*p* < 0.05) higher xylanase production compared to other isolates (Figure 1). The xylanase activity of PF7 was found to be 84.61 ± 3.09 U/ml after 5 days of incubation followed by 82.58 ± 3.61 U/ml for BAG1 after 7 days of incubation under submerged fermentation. The yield of xylanase by PF7 and BAG1 was not significantly (*p* > 0.05) different but the incubation time for maximum xylanase production was more compared to PF7. Whereas the enzyme activity was found to be less for other strains like WD1 and PF4 which produced 42.52 ± 2.76 U/ml after 7 days of incubation and 46.34 ± 2.01 U/ml after 6 days of incubation, respectively.

### Characterization of Fungal Isolate PF7

**FIGURE 2:**
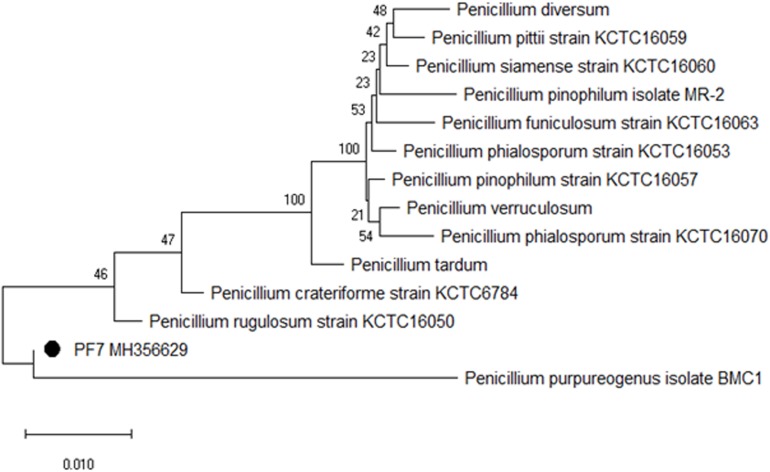
Phylogenetic dendrogram showing the relative position of *P. purpurogenum* PF7 isolated from rotten wood samples and sequence similarity analysis of 5.8S rRNA.

The selected fungal isolate was characterized based on 5.8S rRNA sequencing with reference to ITS1 and 4 regions. The contig sequence of the isolate was subjected to NCBI BLAST to search for homology sequence similarity with existing sequences in the database. Based on the similarity results, the fungal isolate PF7 was identified as *P. purpurogenum* showing 99% sequence similarity. The sequence of the isolate was submitted to the NCBI Genbank with accession no. MH356629. Phylogenetic dendrogram of *P. purpurogenum* was constructed to know the molecular evolutionary relation (Figure 2).

### Optimization of Process Parameters for Xylanase Production by *Taguchi* Methodology

In general the growth conditions for each organism are different and may be they are strain specific. In the current study, we have performed preliminary trials to know the suitable conditions for the growth of *P. purpurogenum*. Further we have selected the conditions like temperature 30°C; pH, 5; moisture, 70%; inoculum, 1.2 × 10^8^ spores/ml; nitrogen source 1% and 7 days of incubation for fermentation which resulted in 376 ± 23.2 U/gds. The same parameters when optimized (temperature 40°C; pH, 3; moisture, 60%; inoculum, 1.2 × 10^8^ spores/ml; nitrogen source 0.8% and 7 days of incubation) by least square methodology, using orthogonal array with L25 design, resulted in 1060 ± 30.0 U/gds which was found to be 64.52% higher (Table 3). The main effects of the factors at their assigned levels on the yield of xylanase by *P. purpurogenum* were shown in Table 4.

**TABLE 4:** Response of each factor on the production of extracellular xylanase by *P. purpurogenum*.

Factor	Level 1	Level 2	Level 3	Level 4	Level 5
Temperature	600.273	580.913	682.600	801.240	280.327
pH	645.593	582.853	525.487	638.473	552.947
Inoculum size	559.133	623.887	519.340	671.133	571.860
Moisture	536.640	649.193	656.840	497.447	605.233
Incubation time	570.680	584.007	625.320	549.880	615.467
Peptone	514.333	603.740	622.827	610.780	593.673

*The enzyme concentrations are expressed in U/gds.*

After optimization of parameters, a separate experiment was carried out with suggested optimum conditions (temperature 40°C; pH, 3; moisture, 70%; inoculum, 1.2 × 10^8^ spores/ml; nitrogen source 0.8% and 5 days of incubation) and the enzyme yield was found to be 1,097 ± 6.76 U/gds which was 3.37% more compared to the yield obtained by run number 16 in the experimental design. The factors chosen for the production of xylanase at their desired level had shown a significant impact on the xylanase yield which was 3.6 times more than un-optimized conditions.

### Status of Biomass During SSF

As the pretreated corn cob residue was the only carbon source used in the present study, its utilization was evaluated based on decrease in weight during the fermentation process. The weight of the biomass has considerably decreased from 5 to 1.6 g within 3 days of incubation and after that again the weight was found to increase to 1.87 g.

### Enzyme Hydrolysis of Alkali Pretreated Corn Cob Residue

The saccharification efficiency of xylanase produced by *P. purpurogenum* has been evaluated by taking both pretreated and raw corn cob as the substrates to investigate the importance of pretreatment on hydrolysis. Enzyme loads with respect to xylose yield and incubation time was optimized. Maximum saccharification of hemicellulose (58.88%) was found with the pretreated substrate where the enzyme load was 2,000 U/gds resulted in 183.42 ± 4.13 mg/g xylose in 48 h of reaction time. Along with xylose, 73.42 ± 3.21 mg/g glucose was produced and the total reducing sugars were amounting to 298.32 ± 9.57 mg/g. Whereas the saccharification of raw corn cob residue resulted in 79.37 ± 0.86 mg/g of total reducing sugars including 41.32 ± 1.23 mg/g and 23.32 mg/g of xylose and glucose, respectively, in 48 h which was significantly low (*p* < 0.001) compared to the pretreated biomass saccharification suggesting the importance of pretreatment.

### Ultrasound Improvement of Enzyme Saccharification

In order to improve the enzymatic hydrolysis of pretreated corn cob residue, the effect of ultrasound on xylanase activity was studied. With the ultrasound vibrations, the rate of enzyme reaction was reduced from 48 to 6 h with 12.02% increase in xylose yield. The reaction time was significantly reduced suggesting the impact of sonic waves on the rate of reaction. In a reaction period of 6 h, 209.67 ± 3.79 mg/g xylose was obtained which was significantly (*p* < 0.05) high.

## Discussion

From the results of chemical composition, it was evident that corn cob contains significant proportions of both cellulose and hemicellulose which make the feedstock a sustainable alternative for biofuel production. Apart from these two polymers, it also contains 18.4% lignin which makes the substrate recalcitrant for use. To overcome this hurdle of recalcitrance, the biomass was pretreated with dilute alkali which increased the surface area of the biomass and made it more accessible for degradation. Hemicellulose is majorly composed of xylan, which acts as inducer for the production of xylanase. Therefore, lignocellulosic feedstocks with high hemicellulose content like corn cobs prove to be a suitable substrate for the production of xylanases. The xylanase yield was increased by 3.6 times with the optimization of parameters for SSF, which underline the significance of each factor in combination with other factors on the production of xylanase by *P. purpurogenum*. Whereas this could not have been possible with conventional method where the effect of individual factor was studied. The optimized conditions that were specifically responsible for the positive regulation of metabolism for higher xylanase production were selected using the optimization tools.

Among the six factors selected for the study, the maximum and minimum contributions on the enzyme yield were 73 and 2% with temperature and incubation time, respectively. The xylanase yield was significantly dropped from 801.24 to 280.38 U/gds when the temperature increased from level 4 to 5 showing its significant impact (Table 4). This is in contrary to the reports of Lakshmi et al. (2009) where the temperature had a minimum impact of 4.67% on the production of xylanase by *Aspergillus terreus* using palm industrial waste. Another study reported that the change in moisture content was an important factor with 24.93% whereas temperature had 17.26% impact on xylanase production by *Trichoderma longibrachiatum* using a mixture of wheat bran and wheat straw (Azin et al. (2007). In the present study, the concentration of peptone also had shown least impact with 4%, therefore minimum concentration of peptone can be used to reduce the process cost. The highest yield was found with experiment run 16, where the peptone concentration was 0.8% and the lowest yield was with run number 23 where the peptone concentration was 1.2%. Notably, despite the increase in the concentration of nitrogen source there was a decrease in the yield suggesting the plausible influence of remaining critical factors on the yield. The contribution of pH on the final yield was 5% that implies the production of xylanase by *P. purpurogenum* at wide pH ranges from 3 to 7. The present findings are in contrast to Rani et al. (2014) who reported 16.95% of pH impact on xylanase production by *Thielaviopsis basicola* MTCC 1467 using submerged fermentation. The study suggests that each organism behaves differently at different environmental conditions. Every single organism will have different and definite metabolism which differs even when a physical or chemical parameter gets altered. Every organism requires specific set of parameter to grow optimally and to get the desired product with higher yield. The overall analysis reveals the robustness of *P. purpurogenum* to produce xylanase at varied range of conditions without being significantly affected unless temperature was maintained optimally. Understanding the influence of each parameter and their specific level is highly desired to make the overall fermentation process successful. ANOVA was performed to analyze and determine which parameter is statistically significant. From the calculated data and F ratios, it was observed that all the factors and their level of interactions considered for the study were statistically significant at 95% CI (Confidence interval), indicating all the variability of analysis data expressed with respect to significant effects.

From the mean values of effects of each factor at the specific levels, it was found that the maximum enzyme production was with the level 4 of factor A, i.e., 40°C where 801.24 U/gds of xylanase was produced and minimum was found with level 5 (280.33 U/gds). The results suggest that optimum temperature is one of the key factors for an organism to grow, metabolize and produce desired product (Thanapimmetha et al., 2012) at significant level. For most of the fungus including *Penicillium* sp. the optimum temperature is 30°C and outside that mesophilic range, there could be decrease in the product yield which in contrast to present report where the optimum temperature was found to be 40°C for enzyme production (Saha and Ghosh, 2014). This might be the reason for low yield of xylanase with un-optimized conditions where the temperature was maintained at mesophilic range (30°C). Whereas, according to Steiner et al. (1994) the optimum temperature for cellulase production by *P. purpurogenum* was found to be 28°C using wheat bran as substrate suggesting that all the conditions for the growth and metabolism are strain specific. The effect of pH was found to be prominent at level 1 (pH 3), suggesting that acidic conditions are preferred by *P. purpurogenum* for the production of xylanase. The enzyme production was more at level 4 of inoculum size and thereafter drastically decreased at level 5 that might be due to increase in competition for nutrients with increased inoculum. When the moisture level was considered, with the increase in percentage of moisture from levels 1 to 3, the xylanase production was increased and immediately declined at level 4. The increase in moisture content affects the oxygen diffusion into the solid matrix by blocked pores and thereby affecting the growth of the organism. The incubation time has resulted in gradient incline in the production of enzyme from the levels 1 to 3 and again decreased thereafter. Whereas the peptone concentration has shown significant increase in the enzyme titer from the levels 1 to 2 and further from levels 2 to 5 there was slight change in the yield with maximum yield at level 3. The optimum parameters for each level are shown in the Table 5. The optimization studies could depict the influence of each level of factor on xylanase production by *P. purpurogenum*.

**TABLE 5:** Optimized levels of parameters suggested by the statistical design.

Factors	Level	Level description
Temperature	4	40°C
pH	1	3.0
Inoculum size	4	1.2 × 10^8^
Moisture	3	70%
Incubation time	3	5 days
Peptone	3	0.8% (w/v)

Much literature has been produced on the impact and importance of optimization study to bring out the suitable conditions for an organism to get the desired metabolite. In the present study, maximum xylanase activity was found to be 1060 ± 30.0 U/gds which is in agreement with Sadaf and Khare. (2014) who have reported 1,025 U/gds of deoiled Jatropha curcas seed cake with the optimized conditions under solid state fermentation by *Sporotrichum thermophile*. Whereas Levin et al. (2008) reported 780 U/gds of poplar wood based solid media with malt extract as the nutrient supplement, under SSF using white rot fungi, *Trametes trogii*. Furthermore, Camassol and Dillon (2009) reported maximum xylanase activity of 9.95 ± 1.53 U/gds of bagasse and wheat bran in combination under solid state fermentation using *Penicillium echinulatum*. Apart from optimizing strategies, for the enhanced production of hemicellulolytic enzymes, cloning and expression studies have also been carried out to increase the potential enzyme secretion for lignocellulose degradation to valued products (Mardones et al., 2018; Echeverria and Eyzaguirre, 2019).

**FIGURE 3:**
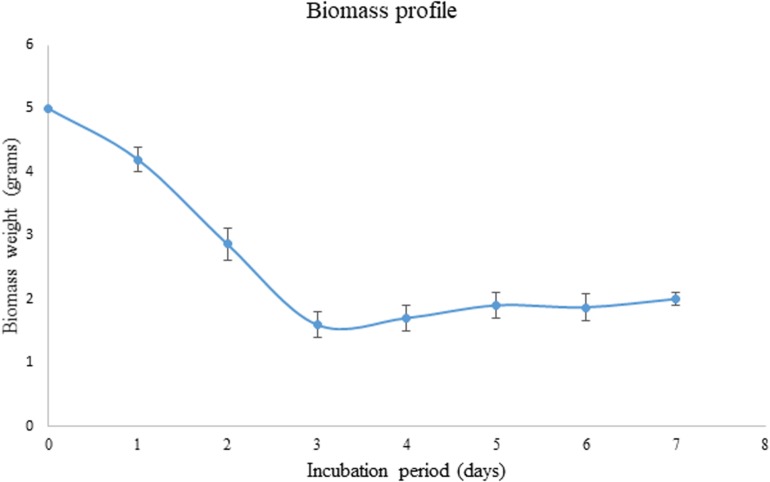
Biomass profile showing the utilization of substrate as carbon source by *P. purpurogenum* during SSF for the xylanase production. Error bars correspond to standard deviation using three replicates.

**FIGURE 4:**
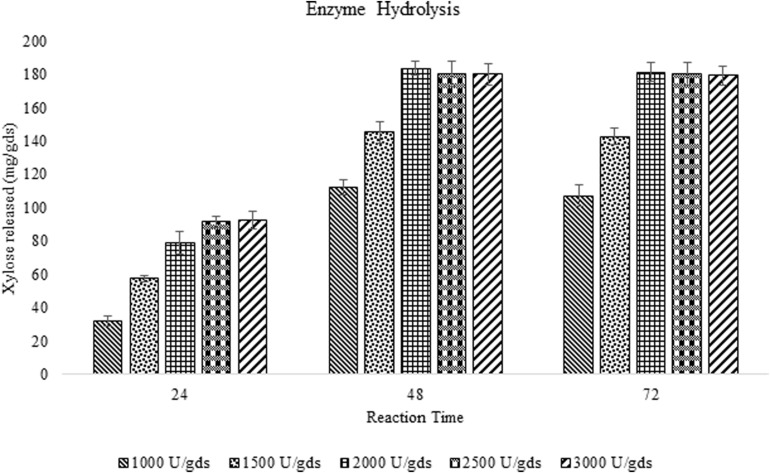
Enzyme saccharification of pretreated corn cob residue and evaluation of efficiency of crude enzyme loads (U/gds) with respect to the yield of fermentable sugars and reaction time (h).

During the fermentation process, the weight of biomass has been logarithmically decreased from days 1 to 3 and again increased thereafter. It has been noticed that 68% of the biomass was reduced within 3 days indicating the logarithmic growth phase of the fungus where the biomass was rapidly utilized for its growth and metabolism (Figure 3). After the day 3, the weight of the biomass was slightly increased and that might be due to the increased growth of fungal mycelium. From the present results it is indicated that the biomass has been adequately utilized as the carbon source for the growth and metabolism of *P. purpurogenum* for the xylanase production. This is in accordance with the findings of Azin et al. (2007) who reported 64% biomass utilization by *Trichoderma longibrachiatum* within 2 days of solid state fermentation using wheat bran in combination with wheat straw. Corn cobs were found to have significant proportion of hemicellulose which is more complex when compared to cellulose and hence requires a complete enzyme cascade for the hydrolysis process. Therefore, rather going for purification of the enzyme mixture, crude enzyme mix was used in the present study for the saccharification process. To evaluate the potential of enzymes, the concentration of crude enzyme and the reaction time was optimized with respect to the release of fermentable sugars. From the results it was found that, boost in xylose concentration in 48 h was dose dependent up to a certain level. The concentration of xylose was exponentially increasing till the enzyme load of 2,000 U/g substrate where maximum fermentation efficiency was 58.88% in 48 h of reaction time with the pretreated substrate (Figure 4). Further, there was no significant change in the sugar yield though the enzyme concentration was increased and this might be due to the saturation of substrate concentration. Therefore, the concentration of substrate and the enzyme need to be at specific ratio to make the enzyme substrate reaction productive.

Furthermore, to investigate the impact of recalcitrance of raw biomass on enzymatic saccharification, pretreated biomass along with raw biomass was used in the enzyme saccharification study. The results clearly imply the impact and importance of pretreatment of biomass on saccharification efficiency and thereby much consideration is being given to the pretreatment of biomass. The pretreatment makes the biomass more accessible which ultimately reflects the yield of the fermentable sugars.

The type and concentration of monosaccharide produced in enzyme reaction could be influenced by the concentration of cellulose and hemicellulose in the biomass (Couturier et al., 2018) along with the efficiency of enzymes released. Maximum release of xylose in the reaction mixture can be justified by the higher concentration of hemicellulose in the corn cob and also the efficiency of *P. purpurogenum* in enzyme production. The glucose concentration was found to be significantly less compared to xylose, though the proportion of cellulose was high. This could be due to considerably low level of cellulase enzyme compared to xylanase in the enzyme cocktail. The present study is in agreement with the reports of Chapla et al. (2010) who reported the production of 172.66 mg/g of reducing sugars using alkali pretreated corn cobs for the enzymatic hydrolysis. Whereas Garai and Kumar (2013) have reported 190.66 mg/g xylose from 15% ammonium pretreated corn cob using the crude enzyme from *Aspergillus candidus*. The variation in the production ratios of fermentable sugars with different feedstocks suggest that every substrate has a different orientation of the linkages in the cellulose and hemicellulose matrix that can react differently with different enzyme cocktail. The concentration of each enzyme in the enzyme cocktail produced by specific organism will be different under the different environmental conditions.

**FIGURE 5:**
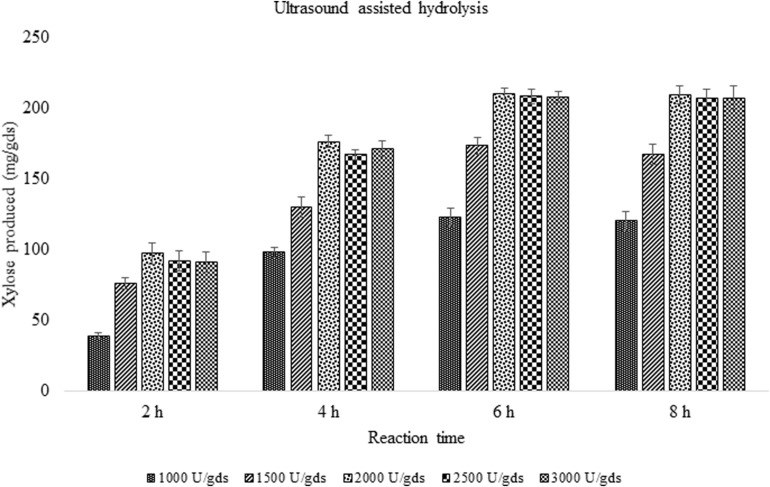
Effect of ultrasound on enzyme saccharification of pretreated corn cob residue by crude enzyme extract obtained (U/gds).

With the aim to improve or intensify the enzyme saccharification, ultrasonic waves were applied and the results were found to increase the yield up to 12.02% with the production of 209.67 ± 8.08 mg/g xylose and 356.71 ± 7.81 mg/g of total sugars. The present findings are in agreement with the earlier reports where the impact of ultrasound on cellulase complex obtained from Novozymes was studied in the enzymatic saccharification of sugarcane bagasse which produced 260 mg/g total sugars (Lunelli et al., 2014). Furthermore, the ultrasound application has reduced the reaction time making it 8 times faster, which is of significant value in the enzyme technology (Figure 5). The ultrasonic waves produced in the reaction mixture, create vapors in the cavities, which on dissociation, form free radicles that speed up the chemical reaction (Rokhina et al., 2009). The study has investigated the impact of ultrasound on xylanase activity and the reaction time which was considerably decreased. The potential of ultrasound in processing aid will be of great value to achieve the higher product yield.

As enzyme hydrolysis is associated with high cost, low product yield and high reaction time, our study aimed to develop productive enzymatic hydrolysis of hemicellulose. The production potential of xylanase by *P. purpurogenum* was optimized to 65% more yield. The same xylanase was used for saccharification of the pretreated corn cob to produce xylose. Apart from a high yield of xylose, the enzyme reaction time was reduced by eight times using ultrasound along with a 12.02% increase in xylose yield. The primary outcome of the study is easy enzyme production that resulted in increased sugar yield in reduced time which has a prominent role in techno-economic biomass conversion and enzyme technology. The bioconversion process not only helps in value addition to agro-waste but also helps in waste management in a sensible way.

## Conclusion

The present investigation involves isolation of an efficient hemicellulolytic strain *P. purpurogenum* from rotten wood by standard screening procedures followed by optimization of critical parameters for the production of extracellular xylanase under SSF. The xylanase yield with optimized conditions was 65.72% higher compared to conventional conditions suggesting the valid impact of our strategy. From the study, it can be deduced that the strain *P. purpurogenum* is considered to be significant with the ability to produce xylanases of potential value. The alkali pretreatment of corn cob had a noteworthy role in the conversion of biomass to valued products. Application of enzyme cocktail with ultrasound assistance released a significant proportion of fermentable sugars from alkali pretreated corn cob residue and has significantly reduced the reaction time which is of great value in enzyme technology.

## Data Availability Statement

The datasets generated for this study are available on request to the corresponding author.

## Author Contributions

All the experiments were designed and carried out by BS with the help of BK. BB designed the concept, monitored all the experiments, and drafted the manuscript. All authors read and approved the final manuscript.

## Conflict of Interest

The authors declare that the research was conducted in the absence of any commercial or financial relationships that could be construed as a potential conflict of interest.

## Footnotes

1 www.ncbi.nlm.nih.gov
